# Biosurfactant and Degradative Enzymes Mediated Crude Oil Degradation by Bacterium *Bacillus subtilis* A1

**DOI:** 10.3389/fmicb.2017.00193

**Published:** 2017-02-09

**Authors:** Punniyakotti Parthipan, Elumalai Preetham, Laura L. Machuca, Pattanathu K. S. M. Rahman, Kadarkarai Murugan, Aruliah Rajasekar

**Affiliations:** ^1^Environmental Molecular Microbiology Research Laboratory, Department of Biotechnology, Thiruvalluvar UniversityVellore, India; ^2^School of Aquatic Food Products and Technology, Kerala University of Fisheries and Ocean StudiesKochi, India; ^3^Curtin Corrosion Engineering Industry Centre, School of Chemical and Petroleum Engineering, Curtin University, BentleyWA, Australia; ^4^School of Science and Engineering, Technology Futures Institute, Teesside UniversityMiddlesbrough, UK; ^5^TeeGene Biotech Ltd., Wilton CentreRedcar, UK; ^6^Division of Entomology, Department of Zoology, School of Life Sciences, Bharathiar UniversityCoimbatore, India; ^7^Thiruvalluvar UniversityVellore, India

**Keywords:** biosurfactant, petroleum remediation, biodegradation, *Bacillus subtilis*, lipopeptide

## Abstract

In this work, the biodegradation of the crude oil by the potential biosurfactant producing *Bacillus subtilis* A1 was investigated. The isolate had the ability to synthesize degradative enzymes such as alkane hydroxylase and alcohol dehydrogenase at the time of biodegradation of hydrocarbon. The biosurfactant producing conditions were optimized as pH 7.0, temperature 40°C, 2% sucrose and 3% of yeast extract as best carbon and nitrogen sources for maximum production of biosurfactant (4.85 g l^-1^). Specifically, the low molecular weight compounds, i.e., C_10_–C_14_ were completely degraded, while C_15_–C_19_ were degraded up to 97% from the total hydrocarbon pools. Overall crude oil degradation efficiency of the strain A1 was about 87% within a short period of time (7 days). The accumulated biosurfactant from the biodegradation medium was characterized to be lipopeptide in nature. The strain A1 was found to be more robust than other reported biosurfactant producing bacteria in degradation efficiency of crude oil due to their enzyme production capability and therefore can be used to remove the hydrocarbon pollutants from contaminated environment.

## Introduction

Environmental pollution due to hydrocarbons, chemicals, solvents and heavy metals are very serious issues that the current world is facing. They are really harmful to living organisms including human beings and also indirectly contribute to the economic losses in developing countries (Ismail et al., 2013). Few of these toxic compounds and xenobiotics including crude oil were naturally degraded to the extent by indigenous microorganisms through biodegradation processes (Hassanshahian et al., 2012). By the biotechnological approach, these pollutant degrading microbes can be identified and effectively used to remove the said contaminants under controlled conditions to produce value added products. Crude oil is one such important pollutant that contains a mixture of low-high molecular weight (MW) hydrocarbons including aromatics, alkanes, asphaltenes and resins in composites (Kumari et al., 2012). Although the number of physicochemical and biological methods exist in literature to remediate the contaminations (Hu et al., 2010), bioremediation is one of the best approaches, since it is more efficient, eco-friendly and cost effective than other methods (Ismail et al., 2013).

Bioremediation is mainly exploiting the biological agents, i.e., bacteria, fungi, or algae, to remove the targeted hydrocarbons. One of the main factors that influence the bioremediation process is the hydrophobic nature of the hydrocarbons. However, the native microbes isolated from hydrocarbon contaminated environments are expected to be more robust in degradation than non-native species. The native microbes can produce metabolites that can easily solubilize the hydrocarbons or other similar pollutants to make them readily available for microbial conversion; therefore, they outperform the non-native or un-adapted cultures (Noparat et al., 2014). Alternatively, co-cultivation of native microbes along with efficient oil degrading microbes is considered a good strategy to increase the contaminant removal in short period of time (Zhang et al., 2012).

Biosurfactants are chemically active surface compounds synthesized by specific groups of microbes that utilize different substrates like simple sugars, oils, hydrocarbons from contaminated environment. They have the ability to reduce surface and interface tension amongst liquid and solid substances and leads to diffuse them as emulsions in liquids (Das and Mukherjee, 2007a). Biosurfactants are widely used for various purposes such as food processing industry, oil recovery process, crude oil drilling lubricants, cleaning purpose, and bioremediation of oil contaminated sites (Makkar et al., 2011; Freitas de Oliveira et al., 2013). Compared to chemical surfactants, biosurfactants have potential advantages, i.e., they are eco-friendly, easily degradable, active in any extreme conditions like high salinity/temperature regions and can be produced using cheap organic sources, which facilitates commercialization (Diaz De Rienzo et al., 2016). Many recent studies report the application of biosurfactant producing microbes in the petroleum contaminated environments to remove hydrocarbon and remediate the environment (Ibrahim et al., 2013; Ferradji et al., 2014). Degradation of hydrocarbons in the presence of microorganisms is enhanced by the production of biosurfactant (Ferradji et al., 2014). Many researchers have identified that *Bacillus* species are potential biosurfactant producers, biodegrading microbes and widely used, e.g., like in microbial enhanced oil recovery (MEOR) (Al-Bahry et al., 2013; Al-Wahaibi et al., 2014), bioremediation purposes (De Franca et al., 2015) and biodegradation (Sakthipriya et al., 2015a). Recently Freitas de Oliveira et al. (2013) extracted the stable biosurfactant from *Bacillus subtilis* for industrial applications. Hence, the biosurfactant plays an important role in bioremediation of hydrocarbon polluted environment.

*Bacillus subtilis* used in this study has been shown to have the highest capability to degrade hydrocarbon by synthesizing biosurfactant in the presence of crude oil as carbon source. However, the production of biosurfactants at larger level still represent a challenge, due to the low production level, low activity, and long fermentation conditions. The biosurfactant production should be improved at industrial level, using efficient microbial strains with higher activity. The optimization of production medium with replacement substrates, the improvement of the efficiency of recovery methods and fermentation processes and the development of biosurfactant producing microorganisms, can open the way to their large scale inexpensive production throughout the enlargement of efficient processes (Mukherjee et al., 2006).

An important factor that influences biosurfactant production is the carbon and nitrogen sources. In addition, the optimization of other environmental factors and growth conditions such as pH, agitation, temperature, and oxygen accessibility are of interest to assess biosurfactant production throughout effects on cellular growth (Desai and Banat, 1997).

Biodegradative enzymes play major role in biodegradation of hydrocarbons (Yong and Zhong, 2010). An important mechanism for alkane removal is the oxygenation of terminal methyl group. While alkane-degrading microbes possess multiple genes for alkane hydroxylases, they are highly competent for degrading the extensive range of alkanes (Van Beilen et al., 2002). Alkane biodegradation is commenced by alkane hydroxylase enzyme to transform alkane to alkanols. Three types of enzymes are known to degrade small, medium and high MW alkanes (Van Beilen and Funhoff, 2007). Methane monooxygenase usually hydroxylates small MW alkanes from ranges of C1–C4, whereas medium chain alkanes such as those ranged between C5–C16 are oxidized by the activity of Alk-B gene that encodes enzymes non-heme alkane monooxygenase (Van Beilen et al., 1994). Higher MW alkanes (>C20) are oxidized by many enzymes such as cytochrome P450s, alkane hydroxylase, flavin-binding monooxygenase, among others (Singh et al., 2012). Another key enzyme that plays a lead role in the biodegradation of hydrocarbons is the alcohol dehydrogenase (Mishra and Singh, 2012). Many bacterial strains such as *Pseudomonas* sp. BP10, *Stenotrophomonas nitritireducens* (Jauhari et al., 2014), *P. aeruginosa* PSA5, *Rhodococcus* sp. NJ2 and *Ochrobactrum intermedium* (Mishra and Singh, 2012) were reported to produce degradative enzymes during the biodegradation of hydrocarbons.

The main purpose of this work was to study the optimization, production, and characterization of the biosurfactant produced by the hydrocarbon utilizing bacteria *B. subtilis* A1 and its application for biodegradation of crude oil. The role of the degradative enzymes in biodegradation of the crude oil was studied. In this work, the functional and structural analyses of the biosurfactant were done using infrared spectroscopy and gas chromatography and mass spectrometry (GC-MS), respectively. Residual crude oil in biodegradation study was quantitatively confirmed using GC-MS analysis.

## Materials and Methods

### Microbial Strain and Culture Conditions

**FIGURE 1:**
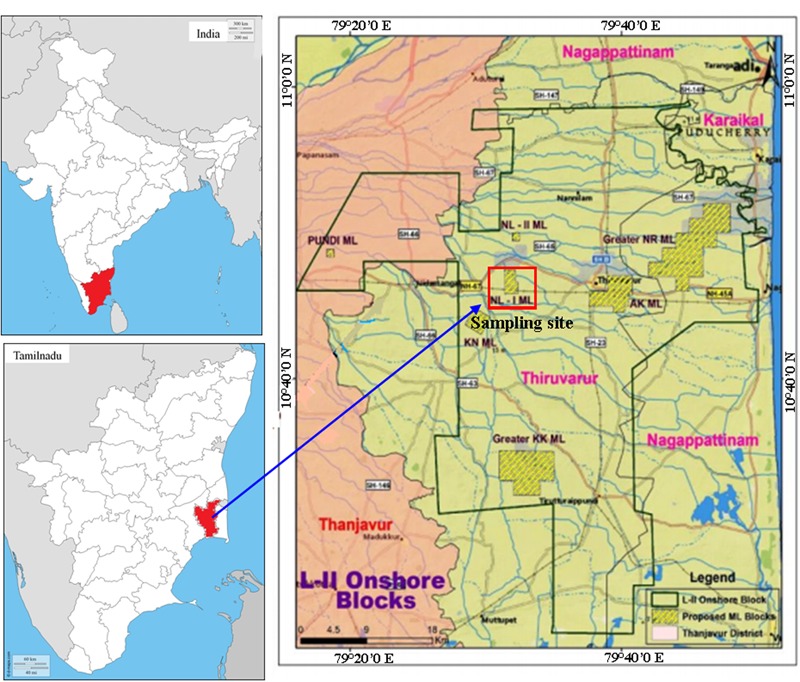
**Map showing the location of sampling sites of crude oil reservoir, Tamilnadu, India**.

In this study bacterium *B. subtilis* A1 was used, which was isolated and identified from an Indian crude oil reservoir also crude oil used in this study was collected from same oil reservoir, the sampling site was presented in **Figure 1** (latitude: 10.6694 and longitude: 79.3155). This strain was identified by 16S rDNA sequencing and deposited under NCBI Genbank accession number KP895564. The strain was retrieved and sub-cultured in Luria–Bertani (LB) agar plates [g/l 10.0 tryptone, 5.0 yeast extract, 10.0 sodium chloride with 15.0 agar (Himedia, Mumbai, India)] and incubated at 37°C for 24 h. Further optimized conditions were applied to culture preparations by single colony inoculation method using LB broth (pH: 7.0) and incubated in an orbital shaker (150 rpm) for 24 h at 37°C.

### Biosurfactant Screening

Biosurfactant production was aerobically carried out in 500 ml Erlenmeyer flask containing 200 ml of sterile Minimal Salt Medium (MSM) (g/l: 0.2 MgSo_4_, 0.02 CaCl_2_, 1.0 KH_2_PO_4_, 1.0 K_2_HPO_4_, 1.0 NH_4_NO_3_, and 0.5 FeCl_3_ Himedia, Mumbai, India), supplemented with 1% (v/v) sterile crude oil (0.22 μm syringe filtered). In triplicate flasks, the pre-culture of *B. subtilis* A1 was inoculated (1.6 × 10^4^ CFU ml^-1^) and incubated at 37°C in an orbital shaker at 200 rpm for 7 days. At the end of the incubation, the biosurfactant was extracted by centrifugation (refrigerated centrifuge, Remi-India: R-248) of culture medium at 4°C for 20 min at 3400 × *g* and the resultant supernatant was utilized for screening purposes. All the assays were performed in triplicate and sterile distilled water was used as the control.

### Drop Collapse Test

Drop collapse test was performed by following the procedure described by Jain et al. (1991) and Patowary et al. (2016) with slight modifications. A drop of crude oil was applied to the glass slide, after that a drop of cell free culture broth was added onto crude oil drop and drop collapse activity was noted. Biosurfactant-producing culture gave flat drops. Deionized water was used as negative control and Triton X-100 (a chemical surfactant) solution used as positive control (1 mg/ml) (Thavasi et al., 2011).

### Oil Displacement Method

Oil displacement technique was carried out as described previously Hassanshahian (2014) 50 ml of distilled water was added to petri dishes followed by addition of 100 μl of sterile crude oil to the surface of the water. Then, 10 μl of the culture filtrate was put on the crude oil surface. The diameter of the clear zone on the oil surface was measured. A negative control was maintained with distilled water (without surfactant), in which no oil displacement or clear zone was observed. Triton X-100 was used as the positive control (Thavasi et al., 2011).

### Emulsification Activity

The emulsification activity of the biosurfactant solutions was determined by measuring the emulsion index (E24) at 25°C as described by Wang et al. (2014). In general, 4 ml of crude oil was poured separately into a test tube containing 4 ml of biosurfactant solution. After being vigorously vortexed for 2 min, the test tube was kept for 24 h and the heights of emulsion, oil and aqueous zones were measured. The emulsion index (E24) was determined as the percentage of height of the emulsified layer (mm) divided by the total height of the liquid column (mm).

### Optimization of Biosurfactant Production

#### Effect of pH

For the optimization of the pH, six different pH were selected namely 5.0, 6.0, 7.0, 8.0, 9.0, and 10.0. MSM was prepared using 1% glucose as sole carbon source and the different pH were adjusted with the help of digital pH meter using 6N HCl and 2N NaOH solutions. After pH adjustment, the medium was sterilized at 121°C for 15 min. Strain A1 (1.6 × 10^4^ CFU ml^-1^) was inoculated and kept at 37°C for 5 days in orbital shaker (150 rpm).

#### Effect of Temperature

Five different temperature were selected for optimization namely, 20, 30, 40, 50 and 60°C. MSM was supplemented with 1% glucose as sole carbon source and pH was adjusted to 7.0 and sterilized at 121°C for 15 min. Strain A1 (1.6 × 10^4^ CFU ml^-1^) was inoculated and kept at the different temperatures for 5 days in an orbital shaker (150 rpm).

#### Effect of Carbon

Carbon substrate plays an important role in biosurfactant production. Eight carbon sources were selected for optimization purposes namely crude oil, coconut oil, diesel oil, sucrose, starch, glycerol, mannitol, and maltose. MSM was prepared with 1% of each carbon source and pH of the medium was adjusted to 7.0, finally sterilized at 121°C for 15 min. Strain A1 (1.6 × 10^4^ CFU ml^-1^) was inoculated and incubated at 40°C for 5 days in orbital shaker (150 rpm).

#### Effect of Nitrogen

Nitrogen is essential for microbial development as well as for effective biosurfactant production. Eight different nitrogen sources were selected for the optimization namely ammonium nitrate, ammonium phosphate, ammonium sulfate, ammonium chloride, peptone, potassium nitrate, yeast extract, and urea. MSM was prepared with each separate nitrogen source (1 g/l) containing 1% of sucrose added as carbon source. The pH of the medium was adjusted to 7.0 and the medium finally sterilized at 121°C for 15 min. Strain A1 (1.6 × 10^4^ CFU ml^-1^) was inoculated and incubated at 40°C for 5 days in orbital shaker (150 rpm).

#### Effect of the Carbon and Nitrogen Concentration

Carbon and nitrogen substrate optimized in this study was further used for optimization of the concentration required for the maximum production. Both optimized carbon and nitrogen sources were added separately in the MSM at different concentration such as: 1, 2, 3, 4, and 5%. Medium pH was adjusted to 7.0 and sterilized at 121°C for 15 min. Strain A1 (1.6 × 10^4^ CFU ml^-1^) was inoculated and incubated at 40°C in orbital shaker (150 rpm) for 5 days. At the end of this study all the optimized parameters such as pH (7.0), temperature (40°C), carbon and nitrogen sources with optimized concentration (2% sucrose and 3% of yeast extract) were set to synthesize biosurfactant by described earlier in this section.

### Analysis for Optimization Conditions and Biosurfactant Extraction

At end of each optimization studies, bacterial cells were removed from surfactant-containing medium by centrifugation using refrigerated centrifuge (Remi-India: R-248) for 20 min at 13,500 × *g* at 4°C and the supernatant was used for the emulsification activity. The optimal growth conditions of the strain were confirmed by emulsification activity and bacterial biomass of each parameter. Bacterial biomass was obtained as described in Santos et al. (2014). Cell free supernatant collected from the optimized study was used for quantify biosurfactant. Crude biosurfactant was obtained as described in Gudiña et al. (2015). In brief, supernatant was acidified to pH 2 using HCl and left for precipitation, precipitated biosurfactant was pooled by centrifugation [refrigerated centrifuge (Remi-India: R-248)] at 7,600 × *g* for 20 min at 4°C. Obtained crude biosurfactant was suspended in double-deionized water and pH was adjusted to 7.0. The biosurfactant solutions were freeze-dried and the products obtained were weighed and stored at -20°C. The surfactant collected in this method was considered as partially purified biosurfactant and used for the characterization purposes.

### Characterization of Biosurfactant

The extracted biosurfactant was further characterized by Fourier transform infrared spectrum (FT-IR) and gas chromatographic mass spectrum (GC-MS) methods. The functional groups of the surfactant collected from *B. subtilis* A1 was qualitatively characterized by FT-IR (Perkin–Elmer, Nicolet Nexus – 470). The dried biosurfactant was ground with the addition of potassium bromide in the ratio of 1:100 and the pellet was fixed in the sample container, and analyzed in the mid IR region 400–4000 cm^-1^. For GC-MS analysis, ∼10 mg of biosurfactant was mixed with 5% HCl-methanol reagent. After the reaction was quenched with addition 1 ml of sterile H_2_O, the sample was recovered with methanol and 1 μl of sample was injected into a gas chromatograph [Shimadzu QP2010 Ultra, Rtx-5Sil MS (30 m × 0.25 mm ID × 0.25 μm)]. The carrier gas used was Helium, the flow rate was set as 1.5 ml min^-1^ and the working temperature of the GC injector was 260°C. The gradient temperature was set as range from 60 to 260°C at a speed of 5°C min^-1^, through an isothermal phase of 10 min at the end of the analysis. The electron impact ion source was sustained at 200°C. Mass spectra were recorded at 70 keV. The mass spectra were obtained with a m/z range: 40–700 ultra-high resolution mode with an acquisition speed of 6 spectra/second. The identification of components was done in scan mode by using NIST11 and Wiley8 library and the target mass spectra obtained from sample were compared with mass spectra obtained from the library.

### Biodegradation of Crude Oil

Biodegradation of crude oil was tested as described by Rahman et al. (2002) with slight changes in incubation period. Pre-cultured *B. subtilis* A1 culture was transferred (initial load about 2.1 × 10^4^ CFU ml^-1^) to a 250 ml of Erlenmeyer screw cap flask, containing 100 ml of MSM with 1% (v/v) filter sterilized crude oil as carbon resource. An un-inoculated control flask was used for monitor abiotic loss of the crude oil substrate. The flasks were incubated at 37°C for 7 days at 200 rpm. Both the experiments were performed in triplicate. Triplicate flasks were recovered from both (inoculated and uninoculated control) systems for every day to measure the growth of total bacterial population, i.e., by conventional serial dilution method using pour plate technique with plate count agar (Himedia, Mumbai, India). For determination of enzyme activity, cells were harvested every day by centrifugation [refrigerated centrifuge (Remi-India: R-248)] at 6000 × *g* at 4°C for 10 min and then used for both enzyme assays.

### Alkane Hydroxylase Activity

Alkane hydroxylase activity during the biodegradation study was confirmed as described in Jauhari et al. (2014). In brief, the collected bacterial cells were rinsed twice and then re-suspended in 2 ml of 20 mM Tris–HCl buffer (pH = 7.4). Bacterial cells were disrupted using sonicator and centrifuged [refrigerated centrifuge (Remi-India: R-248)] at 6000 × *g* at 4°C for 10 min. The cell free supernatant was utilized for testing of alkane hydroxylase activity and absorbance was measured at 340 nm using UV–Vis spectrophotometer (JASCO V-630). 1 ml of testing solution contained 20 mM Tris–Hydrochloride and 0.15% CHAPS buffer (pH 7.4), 0.1 mM of Nicotinamide adenine dinucleotide (NADH), 10 μl of hexadecane mixture (1% hexadecane diluted with 80% DMSO) and 50 μl of crude extract in 1 ml quantity. The reaction was started by adding of 10 μl of hexadecane mixture. The activity of the alkane hydroxylase was expressed as 1 mmol of NADH oxidized per minute.

### Alcohol Dehydrogenase Activity

Alcohol dehydrogenase activity during the biodegradation was measured as mentioned in Jauhari et al. (2014). In brief, cell free supernatant was used for the assay and absorbance was measured at 340 nm using UV–Vis spectrophotometer. 1 ml of reaction solution contained 1 M of Tris– Hydrochloride buffer (pH 8.8), 4 mM of NAD^+^, 100 μl of ethanol (99% pure) and 50 μl of crude extract. Activity of the enzyme alcohol dehydrogenase was recorded as 1 mM of NADH formed per minute.

### Crude Oil Degradation Analysis

Biodegradation of crude oil hydrocarbons was examined by GC-MS analysis. After 7 days of incubation the remaining crude oil present in the culture flask was extracted twice with an equal volume of n-hexane (Adebusoye et al., 2007) and the solvent phase was dried in a vacuum oven at 60°C. 10 μl of resultant crude oil was dissolved in 990 μl of n-hexane. GC-MS model Perkin Elmer, clarus 680, Elite-5MS (30m × 0.25 mm ID × 0.25 μm) was used and 1 μl of sample was injected by split mode at 10:1 ratio. The carrier gas used was Helium, the flow rate was set at 1 ml min^-1^ and the working temperature of the GC injector was 250°C. The gradient temperature was set as range from 60 to 300°C at a speed of 10°C min^-1^, through an isothermal phase of 6 min at the end of the analysis. The mass spectra were obtained with an m/z range: 50–600 ultra-high resolution mode with an acquisition speed of 6 spectra/second. The identification of components was done in scan mode by using NIST08 library and the target mass spectra obtained from sample are compared with mass spectra obtained from the library. The biodegradation of crude oil hydrocarbon was expressed as the percentage (%) of crude oil degraded relative to the quantity of the remaining fractions in the suitable abiotic control samples. The biodegradation efficiency percentage (BE) based on the degradation of hydrocarbons, was calculated as described in Michaud et al. (2004) and Rajasekar et al. (2007). Changes in functional groups of crude oil hydrocarbon during biodegradation were characterized by FT-IR spectroscopy as described in the biosurfactant analysis section.

## Results

### Biosurfactant Screening

The biosurfactant production of the *B. subtilis* A1 was confirmed at the end of the repeated sub-culturing and screening methods and identified *B. subtilis* A1 as an excellent biosurfactant producer. In particular, the strain used in this study gave quick positive results for all biosurfactant screening methods. Specifically, drops collapsed within 30 s indicating higher amount of the biosurfactant present in the solution. Emulsification index was recorded as 76% for initial screening. The results are found consistent with the recent report by Freitas de Oliveira et al. (2013). Biosurfactants produced by different microorganisms are substrate specific, emulsifying diverse hydrocarbons at various rates (Ilori et al., 2005). The present results indicate that biosurfactant produced by *B. subtilis* A1 possess emulsifying activity. In the oil displacement test, a clear zone of ∼2.4 cm was visualized, followed by addition of surfactant solution in the crude oil layer. These results confirmed the presence of biosurfactant in the cell free culture supernatant. After the confirmation of biosurfactant synthesizing capability of the strain *B. subtilis* A1 culture condition was further optimized.

### Biosurfactant Optimization

**FIGURE 2:**
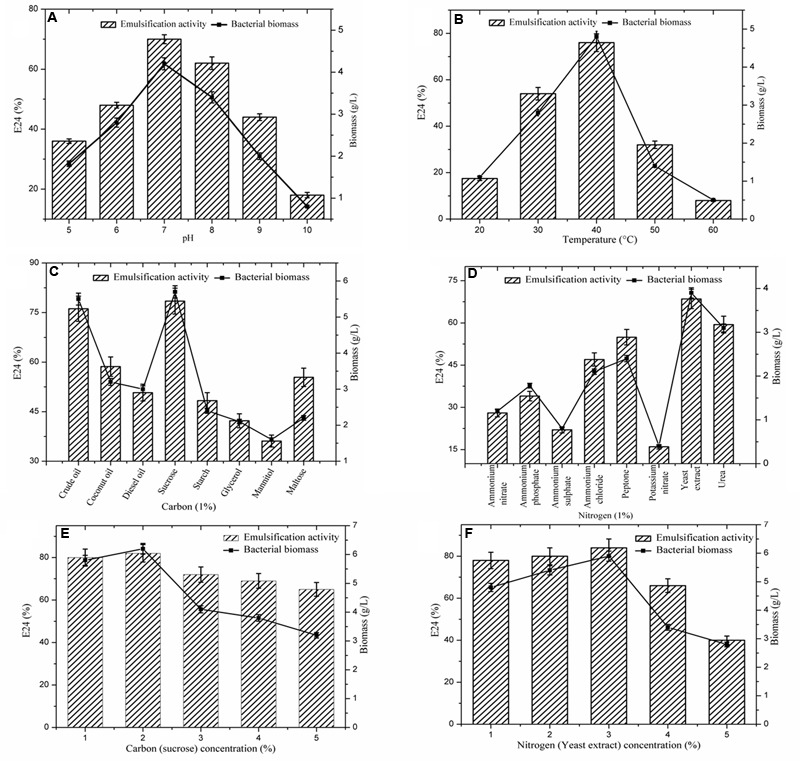
**Effect of different parameters on biosurfactant production. (A)** pH; **(B)** Temperature; **(C)** Carbon sources; **(D)** Nitrogen sources; **(E)** Concentration of carbon; **(F)** Concentration of nitrogen. Vertical bars specify the standard error of the mean based on the three independent tests.

After the initial screening for biosurfactant producing capabilities of the bacterium, *B. subtilis* A1 was further subjected to optimization studies. Five different parameters were selected for optimization studies including pH, temperature and concentration of carbon and nitrogen sources. **Figure 2** shows the optimal parameters obtained for biosurfactant production by strain A1 at the different conditions assessed (Korayem et al., 2015). The synthesis of biosurfactant level was reported in terms of emulsification index (E24%) and cellular activity reported as biomass of the bacterial cells.

Many physiochemical factors such as pH, temperature, growth conditions, and agitation have been shown to strongly influence microbial growth and metabolism (Khopade et al., 2012). Among them, pH of the production medium is a key factor for microbial growth. The optimum pH for the strain A1 was confirmed as 7.0 (E24: 70%), subsequently pH 8.0 showed a considerable effect (**Figure 2A**). Similarly, the role of temperature on biosurfactant production is presented in **Figure 2B**. The optimum temperature was confirmed as 40°C (E24: 76%). Strain A1 is mesophilic bacterium, which indicates this strain exhibits effective production level at moderate temperature (30–40°C).

As represented in the **Figure 2C**, eight carbon sources were screened for biosurfactant production. Among the carbon sources, sucrose was found the most favorable for strain A1 (E24: 78%) (Makkar and Cameotra, 1998; Khopade et al., 2012) followed by crude oil (E24: 76%). Similarly, the effect of the different nitrogen sources on biosurfactant production by strain A1 is presented in **Figure 2D**. Among the eight nitrogen sources, yeast extract showed the highest E24 value (68%) (Kiran et al., 2009; Khopade et al., 2012) followed by Urea (E24: 59%). All the optimized conditions were used to design the production medium. Utilizing the optimal substrate concentrations was essential to determine biosurfactant production. As demonstrated in **Figures 2E,F**, among the given 1–5% of the carbon and nitrogen sources, 2% of the sucrose (E24:.82%) and 3% of the yeast extract (E24: 84%) were found to be the optimum concentrations for biosurfactant production by strain A1. Due to the application of optimized conditions including pH, temperature and carbon and nitrogen sources, E24 values were gradually increased to maximum level in the substrates concentration optimization compared to individual optimization conditions.

Overall, optimized conditions were used for final biosurfactant production as described earlier. Biosurfactant produced by the strain A1 was measured as 4.85 g l^-1^. This found to be maximum and comparable with other literature (Das and Mukherjee, 2007b; De Franca et al., 2015).

### Biosurfactant Characterization

**FIGURE 3:**
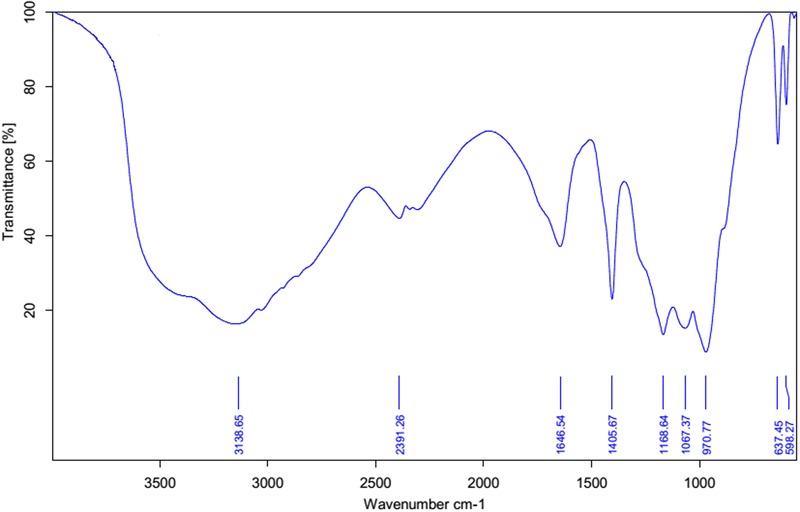
**FT-IR spectrum of partially purified biosurfactant isolated from *Bacillus subtilis* A1**.

Fourier transform infrared spectrum (FT-IR) was recorded for the biosurfactant and revealed the functional groups present (**Figure 3**). The distinctive bands at 3138 cm^-1^ designate the occurrence of -OH bonds (Aparna et al., 2012). The assimilation peak positioned at 1646 and 1168 cm^-1^ states the existence of ester carbonyl groups (-C = O bond in -COOH) (Aparna et al., 2012). The presence of peaks at 2391 cm^-1^ was likely due to the P-H_2_ stretch of phosphines in phosphoserine (Bayoumi et al., 2010). The peak at 1406 cm^-1^ corresponds to aliphatic chains (-CH_3_, -CH_2_-) of the fraction (Pornsunthorntawee et al., 2008). Medium peak was renowned at 970 cm^-1^ shows that presences of O–H bend (carboxylic acids). The absorption peak at 637 cm^-1^ specifies that the presence of -CH_2_ group (Aparna et al., 2012). FT-IR spectra revealed a peak at 598 cm^-1^ arising from C–I (Carbon–Iodine) bond. Based on this observation biosurfactant produced by *B. subtilis* A1 was categorized as lipopeptide in nature (Rodrigues et al., 2006).

**FIGURE 4:**
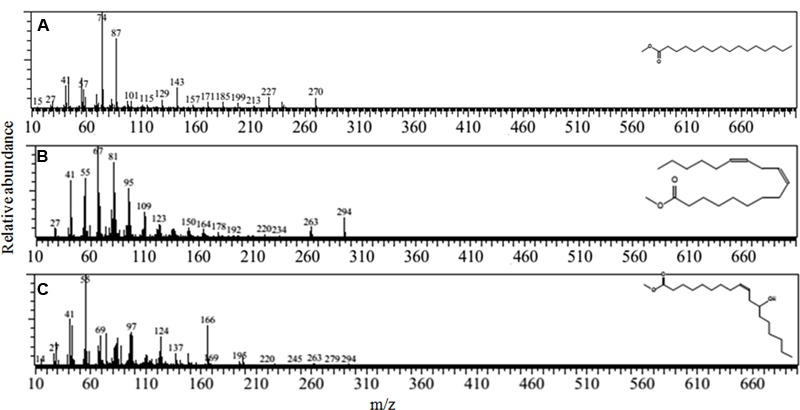
**Mass spectrum of the biosurfactant isolated from *B. subtilis* A1. (A)** Hexadecanoic acid, methyl ester; **(B)** 9, 12- octadecadienoic acid (Z,Z)-, methyl ester; **(C)** 9-octadecenoic acid, 12-hydroxy-, methyl ester.

The gas chromatography and mass spectrum determination further revealed (**Figures 4A–C**) that the biosurfactant extracted from *B. subtilis* A1 was a lipopeptide. Most of the compounds were fatty acids in nature such as hexadecanoic acid, methyl ester (**Figure 4A**) [retention time (RT): 23.14 & 25.48, MW: 270, chemical formula (CF): C_17_H_34_O_2_)] (Kuyukina et al., 2001; Kiran et al., 2010), 9, 12-octadecadienoic acid (Z, Z)-, methyl ester (**Figure 4B**) (RT: 23.94, MW: 294, CF: C_19_H_34_O_2_) (Sadouk et al., 2008), 9-octadecenoic acid, 12-hydroxy-, methyl ester (**Figure 4C**) (RT: 24.71 & 26.35, MW: 312, CF: C_19_H_36_O_3_) (Akintunde et al., 2015). Deshmukh et al. (2012) summarized that the biosurfactant produced by *B. subtilis* was basically lipopeptide in nature.

### Biodegradation of Crude Oil

**FIGURE 5:**
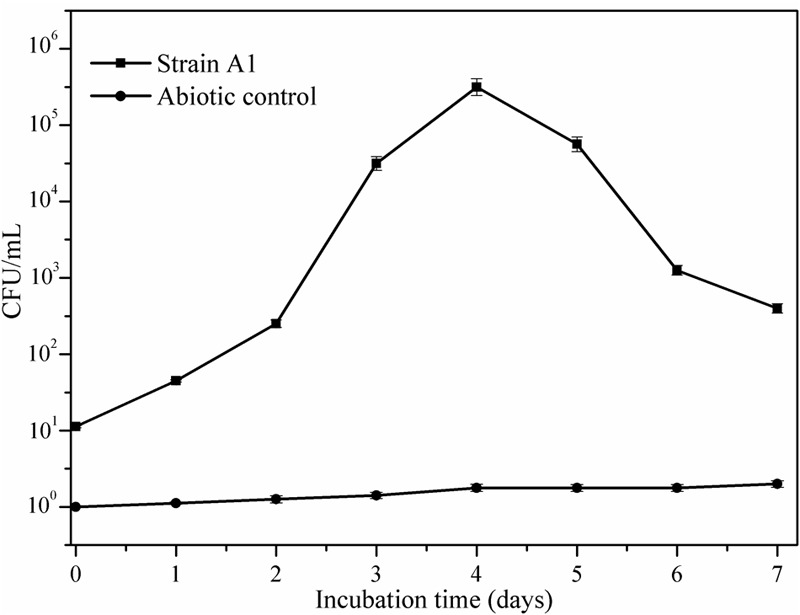
**Bacterial growth curve of *B. subtilis* A1 in MSM with crude oil as a sole carbon source**.

**FIGURE 6:**
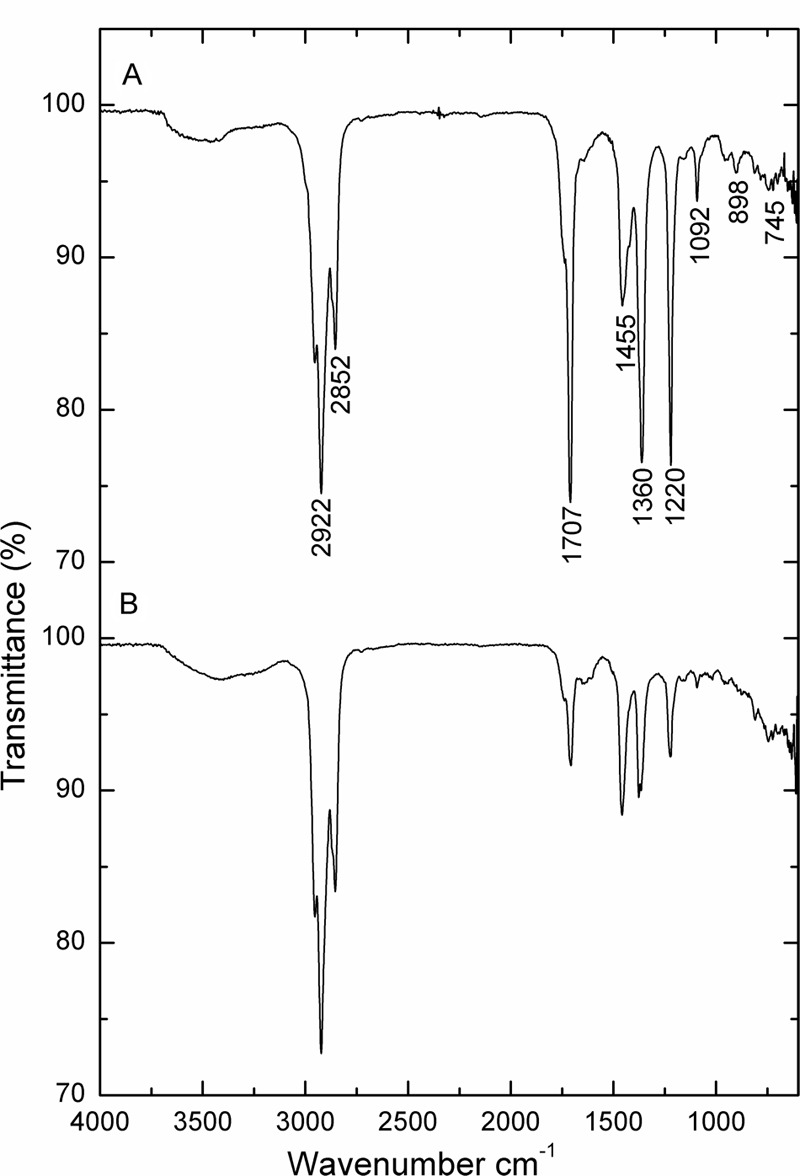
**FT-IR spectrum of crude oil. (A)** Abiotic control system; **(B)** *B. subtilis* A1.

**Figure 5** illustrates the growth status of the isolates in the presence of crude oil as sole carbon source. Utilization of crude oil by biosurfactant producing bacteria was continuously monitored at the time of the biodegradation process. It was visible that the inoculation of *B. subtilis* A1 in MSM broth with crude oil as the sole carbon source turns the medium more turbid within 2 days of incubation. The turbidity of the medium was increased with incubation time. At the end of the incubation period the residual crude oil was recovered and used for further characterization to understand the degradation products. Different functional groups present in the residual crude oil were confirmed by FT-IR spectrum analysis. Both degraded crude oil spectrum and abiotic control spectrum are presented in **Figure 6**. Crude oil in abiotic control (**Figure 6A**), showed distinctive bands at 2922 and 2852 cm^-1^ which belong to C–H aliphatic stretch, a strong peak at 1707 cm^-1^ which is a C = C stretch in aromatic nuclei, medium peaks at 1455 and 1360 cm^-1^ represents the C-H bend for alkanes, sharp and small peaks present at 1220 and 1092 cm^-1^, respectively, correspond to C–N stretch aliphatic amines, presence of peaks at 898 and 745 cm^-1^ is due to the presence of C–H “oop” 2° aromatics. On the other hand, the FT-IR spectrum of degraded crude oil with *B. subtilis* A1 (**Figure 6B**) showed a decrease in the intensity in bands at 1707, 1360, 1220, 1092, 898, and 745 cm^-1^ which indicates degradation of the respective aliphatic and aromatic compounds present in the crude oil.

**FIGURE 7:**
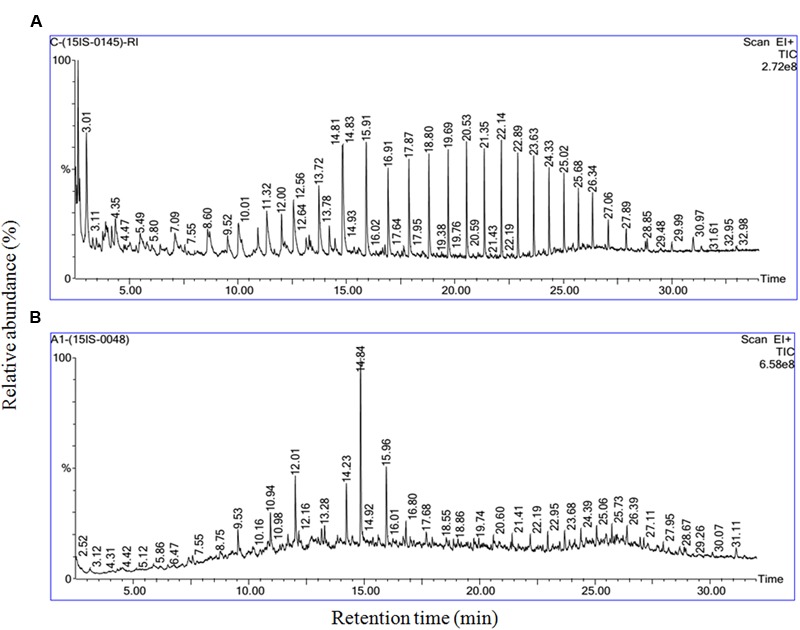
**Gas chromatography and mass spectrometry (GC-MS) characterization of the residual crude oil in crude oil degradation. (A)** Abiotic control system; **(B)** *B. subtilis* A1.

Further degraded sample and abiotic control samples were qualitatively analyzed by GC-MS and were compared in **Figure 7**. The best structural matches of GC retention data of crude oil and mass spectrum interpretation was presented in **Table 1**. **Figure 7A** shows the gas chromatogram of the abiotic crude oil samples. Higher peaks were present in the control and almost all the peaks from control chromatogram disappeared in the experimental samples inoculated with *B. subtilis* A1 (**Figure 7B**). Based on the primary observation it is confirmed that bacteria were capable of utilizing all these hydrocarbon components from the crude oil. The biodegradation efficiency (BE) of the crude oil in presence of *B. subtilis* A1 was calculated to be 87% which was achieved within 7 days. Based on the gas chromatogram analysis it is revealed that crude oil was used as a major carbon source at the hydrocarbon ranges between C_10_–C_29_. More accurately, this bacterial strain completely degraded some of the low MW compounds between C_10_–C_14_. Compounds with ranges of C_15_–C_19_ were degraded nearly 97%, other high molecular compounds are degraded about 78%. This shows that *B. subtilis* A1 has a high capability to degrade the different ranges of alkanes compounds present in the crude oil.

**Table 1:** Biodegradation efficiency (BE) of crude oil the in presence of *Bacillus subtilis* A1.

RT	Compounds	Chemical formula	MW	RA	A1	BE (%)
3.01	Decane, 1-Fluoro-	C_10_H_21_F	160	100	0	100
4.35	Decane, 1-Fluoro-	C_10_H_21_F	160	19	0	100
7.09	Decane, 1-Chloro-	C_10_H_21_Cl	176	11	0	100
8.6	1-Decanol, 2-methyl-	C_11_H_24_O	172	16	0	100
9.52	1-Octanol, 2-Butyl-	C_12_H_26_O	186	13	0	100
10.01	1-Iodo-2-Methylundecane	C_12_H_25_I	296	16	0	100
11.32	1-Iodo-2-Methylundecane	C_12_H_25_I	296	27	0	100
12.56	Dodecane, 2-Methyl-	C_13_H_28_	184	33	0	100
13.72	Decane, 6-Ethyl-2-Methyl-	C_13_H_28_	184	44	0	100
14.83	Dodecane, 4,6-Dimethyl-	C_14_H_30_	198	69	0	100
15.9	Dodecane, 2,6,10-Trimethyl-	C_15_H_32_	212	72	31	57
16.91	Hexadecane	C_16_H_34_	226	55	13	76
17.87	Heptadecane	C_17_H_36_	240	61	5	92
18.80	Octadecane	C_18_H_38_	254	63	2	97
19.69	Heptadecane, 3-Methyl-	C_18_H_38_	254	69	2	97
20.53	Nonadecane	C_19_H_40_	268	75	2	97
21.35	Hexadecane, 2,6,10,14-Tetramethyl-	C_20_H_42_	282	72	8	89
22.14	Heneicosane	C_21_H_44_	296	77	13	83
22.89	Docosane	C_22_H_46_	310	69	11	84
23.63	Tricosane	C_23_H_48_	324	66	11	83
24.33	Tetracosane	C_24_H_50_	338	55	8	85
25.02	Tetracosane	C_24_H_50_	338	50	8	84
26.34	Pentacosane	C_25_H_52_	352	33	11	67
27.06	Hexacosane	C_26_H_54_	366	19	5	74
27.8	Octadecane, 9-Ethyl-9-Heptyl-	C_27_H_56_	380	13	5	62
28.8	Eicosane, 9-Octyl-	C_28_H_58_	394	5	2	60
29.9	Eicosane, 9-Octyl-	C_28_H_58_	394	5	1	80
30.9	Nonacosane	C_29_H_60_	408	8	1	88
**Total biodegradation efficiency (%)**						**87**

*RT, Retention time; MW, Molecular weight; RA, Relative abundance (%).*

### Degradative Enzymes in Biodegradation of Crude Oil

**FIGURE 8:**
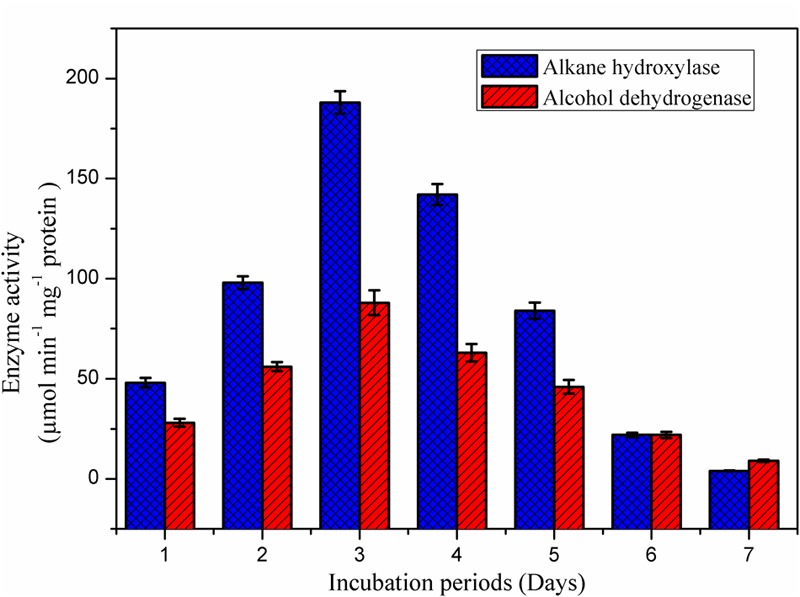
**Appearance of degradative enzyme activity of the *B. subtilis* A1 during biodegradation of crude oil**.

The alkane hydroxylase enzyme was induced in presence of *B. subtilis* A1 during the crude oil degradation (**Figure 8**). Activity of the alkane hydroxylase was increased with incubation period; the maximum activity was recorded as 188 μmol min^-1^ mg^-1^ protein at 3rd day. After that enzyme activity was slowly decreased with incubation period. This level of enzyme activity was much higher than reported in previous studies (Mishra and Singh, 2012). Alkane hydroxylase begins the degradation of alkanes by introducing the oxygen atoms at various sites of alkane terminus (Ji et al., 2013).

As reported by the Mishra and Singh (2012), activity of the alcohol dehydrogenase was not found as high as for alkane hydroxylase during the biodegradation. Activity of the alcohol dehydrogenase was gradually increased toward incubation period (**Figure 8**). The maximum enzyme activity 88 μmol min^-1^ mg^-1^ protein was attained at 3rd day of incubation as similarly recorded for alkane hydroxylase enzyme. After reaching the maximum activity then enzyme production was slowly declined as a decrease of the bacterial growth was observed as mentioned in the **Figure 5**. Similarly Pirog et al. (2010) also reported higher alkane hydroxylase activity compared to alcohol dehydrogenase during biodegradation of hexadecane by *Rhodococcus erythropolis* EK-1.

## Discussion

There are many reports that support the efficiency of *Bacillus* sp. on biosurfactant production and thus they have been widely used for many applications such as in oil recovery process (Pereira et al., 2013), bioremediation purposes (Cubitto et al., 2004; Greenwell et al., 2016), industrial application and degradation purposes (Ismail et al., 2013). Among the used screening methods, oil displacement method was considerably good, since the oil displacement area (clearing zone) in this assay is directly proportional to the concentration of the biosurfactant in the solution (Morikawa et al., 2000). Many researchers have reported the use of these screening methods to study biosurfactant production efficiency (Batista et al., 2006; Ismail et al., 2013).

Most of the bacterial strains are known to exhibit higher activities under optimal growth conditions. Each and every bacterium has optimum pH level for their proficient metabolism; a minute modification in the pH level of the production medium may lead to the complete reduction of the activity. In this study, biosurfactant synthesis was rigorously reduced at lower pH and the bacterial proliferation was considerably impeded. This low pH developed harsh conditions for the bacterium (Khopade et al., 2012). In this study, the starting pH of production medium was set as more than 7, (e.g., pH: 8.0–10.0) biosurfactant production level was declined. Similar results were recorded for other strains, e.g., *Streptomyces* sp (Khopade et al., 2012). Rhamnolipid synthesis using *Pseudomonas* spp. was at its highest production at a pH range of 6–6.5 and decline harshly beyond pH 7.0 (Kiran et al., 2009). Similar to the pH, temperature play a key role in the bacterial activities. A decrease in temperature (for instance, 20°C) makes many bacterial mesophilic strains to slow down their metabolism leading to a reduction in their regular activities. Similarly, higher temperature condition such as 60°C is expected to stop the metabolism of mesophilic bacteria.

Biosurfactants are usually a mixture of complex molecules like peptides, fatty acids, and polysaccharide that have the ability to reduce surface tension through the solubilisation of the fatty acids present in the crude oil, thus leading to proficient exploitation of hydrocarbon by microbes. The growth of microbes on hydrocarbons is habitually related to the development of surfactants (Rajasekar et al., 2008). Biosurfactant production permits the utilization of hydrocarbons by microorganisms, and their succeeding development which has considerable application in the oil industry (Maruthamuthu et al., 2005).

Emulsification activity of the crude oil substrate in this study showed biosurfactant synthesis by the *B. subtilis* A1 (Ismail et al., 2013). Recently Al-Wahaibi et al. (2014) reported about 50% of the emulsification activity with low biosurfactant production (0.5 g/l) by the strain *B. subtilis* B30. Similarly Dastgheib et al. (2008) presented 65% of the emulsification activity by strain *Bacillus* species. Al-Bahry et al. (2013) reported low biosurfactant yield (2.29 g/l) by the strain *B. subtilis* B20. Based on these comparison strain *B. subtilis* A1 was confirmed as efficient biosurfactant producer with higher emulsification activity. FT-IR analysis of the surfactant isolated from *B. subtilis* A1 exposed the existence of nine absorption peaks. All the absorption peaks demonstrated the presence of fatty acids and peptides (Bayoumi et al., 2010). Bayoumi et al. (2010) reported that phosphines presence in the biosurfactant produced by *B. subtilis* strain. Ibrahim et al. (2013) reported that the lipopeptide based biosurfactant contains fatty acids such as octadecanoic acid and 9-octadecenoic acid as major components. Based on the GCMS analysis applied in this study, the predominant biosurfactant compounds are lipopeptide in nature (Ibrahim et al., 2013). Another fatty acid compound hexadecanoic acid was also detected in the biosurfactant (Peng et al., 2008).

The cationic moieties of the biosurfactant attract the negatively charged bacterial membrane in contact with crude oil during degradation (Ferradji et al., 2014). Crude oil is a complex mixture of insoluble compounds, alongside n-alkanes of different chain-lengths, which are hydrophobic and cautiously disperse in water. Synthesis of surface active substances from the degradation of short chain low MW hydrocarbons by microorganisms leads to the beginning of the solubilisation of crude oil and the turbidity of the culture medium. The increase in turbidity could be due to many factors such as cell growth together with emulsification of the oil present in culture media and production of other extracellular molecules (Chandankere et al., 2014). Biosurfactant synthesis is related to cellular development, as an increase in biomass concentration leads to an increase in emulsification activity. In the case of growth-related biosurfactant production there is a parallel correlation between the substrate utilization, microbial growth and biosurfactant production. As a consequence of this, intensifying amounts of crude oil were diffused into the culture medium, leading to a sudden increase in the culture turbidity. Biosurfactant synthesized by bacteria are more proficient than chemical surfactants in increasing the solubility and well-organized biodegradation of petroleum hydrocarbons. They are also eco-friendly in nature (Zeng et al., 2011). In this study, the privileged production of biosurfactant by *B. subtilis* A1 was simultaneous to the consumption of accessible hydrophobic substrates by escalating the surface area of substrates and solubility. Besides strain A1 was identified as efficient crude oil degrader compared to other *B. subtilis* strain. Recently Sakthipriya et al. (2015b) achieved 80% of the degradation efficiency (10 days). Similarly Bezza and Chirwa (2015) reported 82% of the biodegradation efficiency using *B. subtilis* strain after the long incubation period (18 days). Ijah and Ukpe (1992) summarized very low degradation efficiency (44.1–50.4%) using two *Bacillus* species with long incubation period (20 days).

Degradative enzyme producing capabilities of the bacterial strain make them an efficient strain among other. Recently Mishra and Singh (2012), have reported that alkane hydroxylase enzyme play an important role in the degradation of n-hexadecane by bacterial strains *P. aeruginosa* PSA5 and *Rhodococcu*s sp. NJ2. These enzymes play an important role in the hydrocarbon degradation and the respective genes that encode those enzymes were identified in recent studies (Whyte et al., 2002; Hassanshahian et al., 2012).

Both biosurfactant and enzyme production by *B. subtilis* A1 strain led to an increase in the efficiency of biodegradation in the present investigation. Several studies have shown that alkanes ranged between C_14_–C_20_ were easily utilizable as energy source by most of the hydrocarbon degrading bacteria (Sanjeet et al., 2004; Das and Mukherjee, 2007a). In this work we found that more than 97% of the alkanes ranged from the C_15_–C_19_ were utilized by the *B. subtilis* A1, which is due to the production of alkane hydroxylase enzyme during the degradation process. Ibrahim et al. (2013) identified many bacterial genera including *Achromobacter* sp., *Bacillus* sp., *Serratia* sp., *Sphingomonas* sp. and *Micrococcus* sp. as crude oil degrading bacteria and biosurfactant producers. The produced biosurfactant was also described as lipopeptide in nature. Recently Bezza and Chirwa (2015) reported the application of the bacterial strain *B. subtilis* in bioremediation and oil recovery process by production of biosurfactant of lipopeptide nature (Bezza and Chirwa, 2015). The present study confirms that Gram positive *B. subtilis* A1 has the ability to produce biosurfactant of lipopeptide nature which exhibits efficient uptake of hydrocarbons in crude oil.

## Conclusion

*Bacillus subtilis* A1 produced high amounts of biosurfactant and degradative enzymes in presence of crude oil as a substrate. Optimum growth condition was confirmed for maximum biosurfactant production such as pH 7.0, temperature is 40°C, sucrose and yeast extract acted as best carbon and nitrogen sources, respectively. 4.85 g l^-1^ of biosurfactant was produced with optimized conditions and synthesized biosurfactant was lipopeptide in nature and exhibited high emulsification activity. Biodegradation efficiency of the crude oil was 87% which was associated with high production of biosurfactant, alkane hydroxylase, and alcohol dehydrogenase enzymes. This strain completely degraded the low MW hydrocarbons (C_10_–C_14_) and exhibited up to 97% degradation of high MW hydrocarbons range between C_15_–C_19_. These results illustrate that *B. subtilis* A1 is a very efficient crude oil degrading bacterium. The bioavailability of the crude oil hydrocarbons may be credited to its biosurfactant synthesis abilities and emulsification capabilities as well as the key function of the degradative enzymes on the degradation of hydrocarbons. This strain could be used in the bioremediation of crude oil/PAH contaminated environments.

## Author Contributions

All authors listed, have made considerable, direct and logical contribution to the work, and approved it for publication. That is, PP, PR, KM, and AR designed the experiments. PP performed the experiments and drafted the manuscript. EP took contribution in data analysis and interpretation of GCMS samples. LM, PR, and KM revised the manuscript and approved the final version. All authors discussed the results and commented on the manuscript.

## Conflict of Interest Statement

The authors declare that the research was conducted in the absence of any commercial or financial relationships that could be construed as a potential conflict of interest.
